# Blueberries Improve Endothelial Function, but Not Blood Pressure, in Adults with Metabolic Syndrome: A Randomized, Double-Blind, Placebo-Controlled Clinical Trial

**DOI:** 10.3390/nu7064107

**Published:** 2015-05-27

**Authors:** April J. Stull, Katherine C. Cash, Catherine M. Champagne, Alok K. Gupta, Raymond Boston, Robbie A. Beyl, William D. Johnson, William T. Cefalu

**Affiliations:** 1 Pennington Biomedical Research Center, Louisiana State University System, Baton Rouge, LA 70808, USA; E-Mails: katherinecash@gmail.com (K.C.C.); catherine.champagne@pbrc.edu (C.M.C.); robbie.beyl@pbrc.edu (R.A.B.); william.johnson@pbrc.edu (W.D.J.); william.cefalu@pbrc.edu (W.T.C.); 2 Center for the Study of Botanicals and Metabolic Syndrome, Pennington Biomedical Research Center, Louisiana State University System, Baton Rouge, LA 70808, USA; 3 Baton Rouge VA Outpatient Clinic, Southeast Louisiana Veterans Health Care System, 7968 Essen Park Avenue, Baton Rouge, LA 70809, USA; E-Mail: alok.gupta1956@gmail.com; 4 Department of Clinical Studies, New Bolton Center, School of Veterinary Medicine, University of Pennsylvania, Philadelphia, PA 19348, USA; E-Mail: drrayboston@yahoo.com

**Keywords:** blueberries, endothelial function, endothelial dysfunction, prediabetes, hypertension, cardiovascular risk factors

## Abstract

Blueberry consumption has been shown to have various health benefits in humans. However, little is known about the effect of blueberry consumption on blood pressure, endothelial function and insulin sensitivity in humans. The present study investigated the role of blueberry consumption on modifying blood pressure in subjects with metabolic syndrome. In addition, endothelial function and insulin sensitivity (secondary measurements) were also assessed. A double-blind and placebo-controlled study was conducted in 44 adults (blueberry, *n* = 23; and placebo, *n* = 21). They were randomized to receive a blueberry or placebo smoothie twice daily for six weeks. Twenty-four-hour ambulatory blood pressure, endothelial function and insulin sensitivity were assessed pre- and post-intervention. The blood pressure and insulin sensitivity did not differ between the blueberry and placebo groups. However, the mean change in resting endothelial function, expressed as reactive hyperemia index (RHI), was improved significantly more in the group consuming the blueberries *versus* the placebo group (*p* = 0.024). Even after adjusting for confounding factors, *i.e*., the percent body fat and gender, the blueberry group still had a greater improvement in endothelial function when compared to their counterpart (RHI; 0.32 ± 0.13 *versus* −0.33 ± 0.14; *p* = 0.0023). In conclusion, daily dietary consumption of blueberries did not improve blood pressure, but improved (*i.e*., increased) endothelial function over six weeks in subjects with metabolic syndrome.

## 1. Introduction

Cardiovascular disease (CVD) is the leading cause of death in the United States [1]. The risk factors for CVD encompass insulin resistance, central obesity, dyslipidemia and hypertension, which are also features of metabolic syndrome [2]. These risk factors contribute to vascular abnormalities, such as endothelial dysfunction, which represents a very early step in the process of atherosclerosis and is also associated with increased adverse CVD outcomes [3]. Endothelial dysfunction can be improved, and some studies indicate that the risk of developing endothelial dysfunction increases with the number of risk factors present in an individual [4,5].

In the goal to reduce CVD, preventative strategies are clearly a major focus of attention. Epidemiological studies have shown that the consumption of fruits and vegetables is inversely associated with the risk of developing CVD and stroke [6,7], with foods that are higher in polyphenols (specifically anthocyanins) having a lower risk of myocardial infarction [8]. We have previously shown that the consumption of anthocyanin-rich blueberries improved insulin resistance [9], a known CVD risk factor, and this has also been observed in rodents [10]. Additionally, research in Klimis-Zacas laboratory [11,12,13,14,15] along with other research laboratories [16,17,18] has found that blueberries improved blood pressure, another known CVD risk factor, and/or endothelial function in rodents. Furthermore, there are limited data from clinical trials evaluating the effects of blueberries or anthocyanin berry extracts on blood pressure and endothelial function [19,20,21,22,23,24,25], with fewer studies having significant findings for endothelial function [19,20] and/or blood pressure [19,23,25]. Thus far, only clinic blood pressure measurements have been used, and the present study sought to confirm the antihypertensive effect of blueberries using 24-hour ambulatory blood pressure monitoring. Measuring blood pressure over seven days allows for determining the consistency of either normality or abnormality of blood pressure variability over serial consecutive 24-hour spans [26]. Therefore, the primary objective of the present study was to assess whether blueberry consumption was effective at modifying blood pressure in subjects at high risk for developing CVD. The secondary objective was to evaluate the effects of blueberries on modifying endothelial function and insulin sensitivity. We hypothesized that daily consumption of blueberries for six weeks would be effective in improving blood pressure, endothelial function and insulin sensitivity in a population with metabolic syndrome.

## 2. Experimental Section

### 2.1. Subjects

Participants in the study were recruited from the Greater Baton Rouge, Louisiana, area. All participants gave written informed consent before participating in the study. The inclusion criteria included participants who were older than 20 years and met the definition of metabolic syndrome as defined by the World Health Organization [2]. Participants had to exhibit impaired fasting glucose or impaired glucose tolerance and insulin levels ≥5 μIU/mL, plus ≥2 of the following risk factors: obesity (BMI > 30 kg/m^2^), elevated blood pressure (no antihypertensive medication, 140–179 mmHg systolic or 90–109 mmHg diastolic; or antihypertensive medication), elevated triglycerides (≥150 mg/dL) and/or reduced high-density lipoprotein (HDL; men, <35 mg/dL; or women, <39 mg/dL). Subjects with known diseases, such as diabetes, liver, kidney or heart disease, were excluded. Furthermore, subjects that smoked were excluded from the study. The majority of our participants were on antihypertensive medication and were asked to maintain their same medication dosage and usage without any changes during the duration of the study. Furthermore, participants abstained from using nonprescription drugs, vitamins, dietary supplements and herbal supplements two weeks prior to the start of the study and throughout the duration of the study.

### 2.2. Study Design

A six-week randomized, double-blind, placebo-controlled and parallel-arm human dietary intervention trial was conducted at Pennington Biomedical Research Center (Baton Rouge, LA, USA). Recruitment began in July 2010 and continued until June 2012, when the final participant was finished. The study was approved by Pennington Biomedical Research Center’s Institutional Review Board for human subjects (Approval Number 10014) and registered at the United States National Institutes of Health (clinicaltrials.gov; NCT01399138). Participants who met the inclusion criteria were randomized to either the blueberry or matched placebo group and required to visit the clinic weekly over six weeks. Allocation to either the treatment or placebo group was based on a randomization table created by the study biostatistician, and block randomization was used with random functions in SAS 9.3 to ensure well-balanced groups. After the table was generated, the kitchen staff randomized the participants to the treatment or placebo group. The participants and treatment or placebo smoothie were identified only by code numbers. The trial investigators and participants remained blinded until after the study was completed and data analysis was performed.

The participants consumed twice daily a 12-oz yogurt and skim milk-based smoothie with 22.5 g of freeze-dried blueberry powder added (total 45 g/day) or an identical smoothie without the blueberry powder (*i.e*., placebo). The smoothies were identical in both physical appearance (*i.e*., color), taste, macronutrient content (controlled for fiber content) and consistency, with the noted exception of blueberry powder in the blueberry smoothie (Table 1). The dose selection was based on previous clinical trials that observed many health benefits with a similar dose [9,25]. A registered dietitian instructed the participants to consume the first smoothie during breakfast time and the second smoothie during dinner time (at least 6 hours apart). The smoothies were prepared in the metabolic kitchen, and a rolling cooler was packed with a week’s supply of frozen smoothies for the participants to pick-up at their weekly visits. The 45 g of freeze-dried blueberry powder equated to approximately 2 cups of fresh whole blueberries and was prepared and supplied by the United States Highbush Blueberry Council (Folsom, CA, USA). The powder was made from a 50/50 mixture of two varieties of highbush blueberries (Tifblue (*Vaccinium ashei*) and Rubel (*Vaccinium corymbosum*)). The whole blueberries were freeze-dried, milled and stored in aluminum cans under nitrogen. The blueberry powder contained total phenolics (determined with Folin-Ciocalteu reagent by the method of Slinkard and Singleton; gallic acid was used as a standard [27]), anthocyanins (measured using the pH differential method; cyanidin-3-glucoside was used as a standard [28]) and antioxidants (used the oxygen radical absorbance capacity assays; Trolox was used as the reference standard [29]), which were analyzed by Brunswick Laboratories (Southborough, MA, USA; Table 1). Furthermore, the nutritional composition of the blueberry powder was analyzed by Medallion Labs (Minneapolis, MN, USA; Table 1).

**Table 1:** Nutritional composition and ingredients in the smoothies.

Nutritional Composition	Blueberry	Placebo	Ingredients	Blueberry	Placebo
Energy, kcal *	242	241	Yoplait^®^ 99% fat-free creamy vanilla yogurt, g	125	130
Carbohydrate, g	49.7	51	Skim milk, g	105	115
Fiber, g	4.5	4.5	Freeze-dried blueberry powder, g	22.5	—
Protein, g	8.0	7.9	Imitation vanilla flavor, g	5	—
Fat, g	1.1	1.0	Sugar, g	—	14
Saturated fat, g	0.6	0.6	Splenda, g	1	—
Sodium, mg	110.5	114.6	Benefiber, g	—	5.2
Vitamin C, mg	2.7	0	Artificial blueberry flavor (liquid and powder), g	—	5
Vitamin A, IU	764.9	807.3	Red food color, g	—	2.9
Iron, mg	0.22	0.04	Blue food color, g	—	1.1
Calcium, mg	275.3	287.5	Water, g	23.7	9
Antioxidants (ORAC), μmol TE ^#^	6615	—			
Total phenolics, mg	773.6	—			
Anthocyanins, mg	290.3	—			
			Total smoothie weight, g	282.2	282.2

Information is based on one smoothie, and each participant consumed two smoothies per day. The blueberry powder was from the United States Highbush Blueberry Council (Folsom, CA, USA); * 1 kcal = 4.184 kJ; ^#^ ORAC, oxygen radical absorbance capacity; TE, Trolox equivalents.

Three-day food records (*i.e*., two weekdays and one weekend day) were also administered at the beginning and end of the study by a registered dietitian. The food records were analyzed using the Pennington Biomedical Research Center’s Food Diary Program (Pennington Biomedical Research Foundation). Based on their eating patterns and usual intake, the registered dietitian counseled the participants on the importance of maintaining their body weight by eliminating approximately 500 kcal/day from their daily intake to compensate for the energy consumed in the blueberry and placebo smoothies. The participants were cautioned about gaining weight during the study, and their weight was monitored weekly to verify that there was no weight gain. To eliminate consumption of anthocyanin-containing foods and drinks, the subjects were asked to abstain from consuming berries, grapes, juices that contained berries and grapes, and wine during the study. The rationale for these recommendations was to eliminate consumption of anthocyanin-containing foods and drinks. Furthermore, fruit/wine questionnaires were administered to participants occasionally to document their fruit/wine consumption during the study. To monitor compliance, participants were instructed to return unconsumed smoothies when they picked up their next week of smoothies, and this information was recorded by the dietitian.

### 2.3. Endothelial Function and Blood Pressure

Endothelial function was assessed, in the fasted state (10-hour fast; no eating, drinking, or caffeine use), using the non-invasive EndoPAT™ 2000 device (Itamar Medical Ltd.; Caesarea, Israel) pre- and post-intervention. The participants were asked to remain consistent with taking their antihypertensive medication at the same time every day during the duration of the study. Endothelial function was a non-invasive test that involved the recording of the peripheral arterial tone (PAT) signal from the bio-sensors placed on the index fingers of both hands. The signal was recorded before and after inflating a blood pressure cuff on one arm, which temporarily arrested the blood flow to the fingers. The computer software provided by the manufacturer was used to compare the arterial pressure ratio in the two fingers before and after occlusion. Then the reactive hyperemia index (RHI) was calculated, which was a ratio of the average pulse wave amplitude measured over sixty seconds, starting one minute after cuff deflation to the average pulse wave amplitude measured at the baseline. The other arm served as a control, and the ratio was corrected for changes in the systemic vascular tone. RHI has been shown to correlate with the ischemia-induced flow-mediated dilation in the larger brachial artery measured by high-resolution ultrasound and with the gold standard method (acetylcholine infusion in coronary arteries) for the assessment of endothelial function [30].

Resting clinical systolic and diastolic blood pressure was assessed once a week in the fasted state (10-hour fast; no eating, drinking, or caffeine use). Furthermore, the participants were asked to remain consistent with taking their antihypertensive medication at the same time every day during the duration of the study. Furthermore, 24-hour ambulatory blood pressure was monitored over seven days using an automatic blood pressure monitoring device (Tiba Medical, Inc.; Portland, Oregon, USA) pre- and post-intervention. The blood pressure measurements were taken at 30 minute intervals during the day followed by 60 minute intervals during the night [26], and there were no restrictions on their food and beverage consumption before the measurements were taken.

### 2.4. Body Weight, Body Composition and Insulin Sensitivity

Body weight, body composition and insulin sensitivity were all measured after a 10-hour fast (no eating, drinking, or caffeine use). Furthermore, the participants were asked to remain consistent with taking their antihypertensive medication at the same time every day during the duration of the study. Body weight was measured once a week on a digital balance accurate to 0.1 kg with participants wearing a robe without shoes at each visit. Fat-free mass, fat mass and body fat percentage were measured by dual-energy X-ray absorptiometry pre- and post-intervention. Insulin sensitivity was assessed using a modified frequently sampled intravenous glucose tolerance test (FSIVGTT) pre- and post-intervention [31]. After the fasting blood sample (0 minute), a glucose bolus (300 mg/kg) was given intravenously over one minute. Then a bolus of insulin (0.03 units/kg) was given at the 20-minute time point. Blood was sampled at frequent intervals over a 4-hour period (2, 4, 6, 8, 10, 15, 20, 30, 40, 60, 90, 120, 150, 180, 210 and 240) after the start of the glucose injection. Blood samples were used for analyses of serum glucose and insulin. Insulin sensitivity (SI) was calculated by a minimal model technique and MINMOD Millennium software [32].

### 2.5. Biochemical Analyses

Glucose, insulin, total cholesterol and HDL were measured in fasting serum samples obtained pre- and post-intervention. Glucose and insulin were additionally measured in serum samples during the FSIVGTT. Insulin assays were performed by immunoassay with chemiluminescent detection on a Siemens Immulite 200XPi Immunoassay System (Siemens Healthcare Diagnostic, Inc.; Flanders, NJ, USA). Serum glucose, total cholesterol and HDL were measured on the Beckman Coulter UniCell DXC 600 Synchron Clinical System (Beckman Coulter, Inc., Clinical Diagnostic Division; Brea, CA, USA). LDL-cholesterol was based on a calculation (total cholesterol − HDL − (triglycerides/5)).

### 2.6. Statistical Analyses

This study was powered to detect differences between the blueberry and placebo groups. A total of 44 completers would give 80% power to detect a significant (*p* ≤ 0.05) difference in blood pressure. Statistical analyses were performed using the SAS software Version 9.3 (SAS Institute, Inc.; Cary, NC, USA). Data were expressed as the means ± SEM. A samples *t*-test was used to compare the baseline values of the groups (blueberry *versus* placebo) and also the changes in each evaluated parameter during the course of the study between the groups. Changes in RHI and blood pressure were analyzed by using a *t*-test based on least square means from a linear model, including percent body fat and gender. *p*-Values ≤ 0.05 indicated statistical significance.

## 3. Results

**Figure 1:**
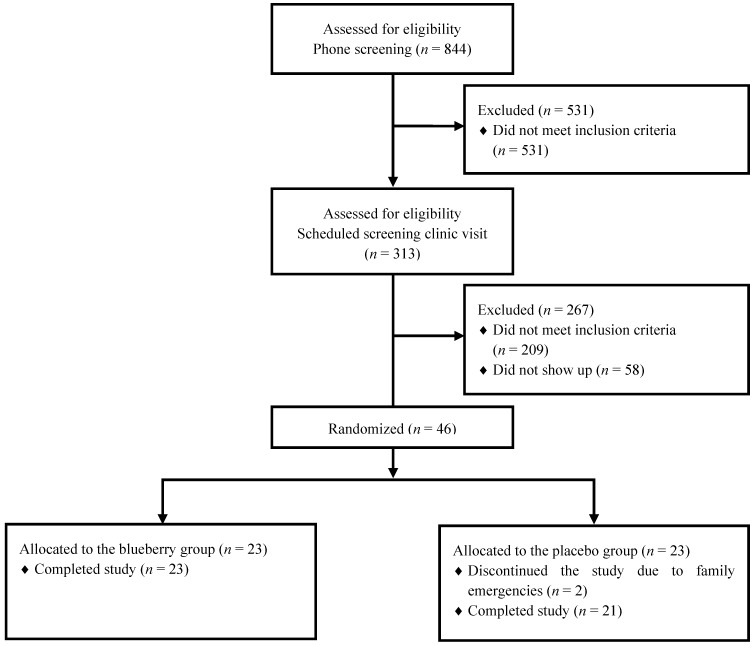
Consolidated Standards of Reporting Trials (CONSORT) flow diagram of participants from recruitment to data analysis.

A total of 46 participants were randomized into the study. Two participants discontinued the study due to family emergencies, and they were in the placebo group (4.3% drop out rate). Therefore, 44 participants completed the study (*n* = 23 blueberry; and *n* = 21 placebo). Figure 1 shows the flow of participants from the phone screening to randomization, and their baseline characteristics are presented in Table 2. The participants were middle-aged and obese with prehypertension (systolic blood pressure, 120–139 mmHg; American Heart Association recommendations) and prediabetes (impaired fasting glucose, 5.6–6.9 mmol/L; American Diabetes Association recommendations). The majority of the blueberry and placebo groups’ baseline characteristics were comparable, except for the percentage of body fat and RHI, which were significantly lower in the blueberry *versus* placebo groups. The mean change from pre- to post-intervention, in the participants’ characteristics, did not differ between the groups (Table 3). Other measured parameters, such as insulin sensitivity, and energy and macronutrient (*i.e*., carbohydrate, protein and fat) intake, did not differ between the groups.

**Figure 2:**
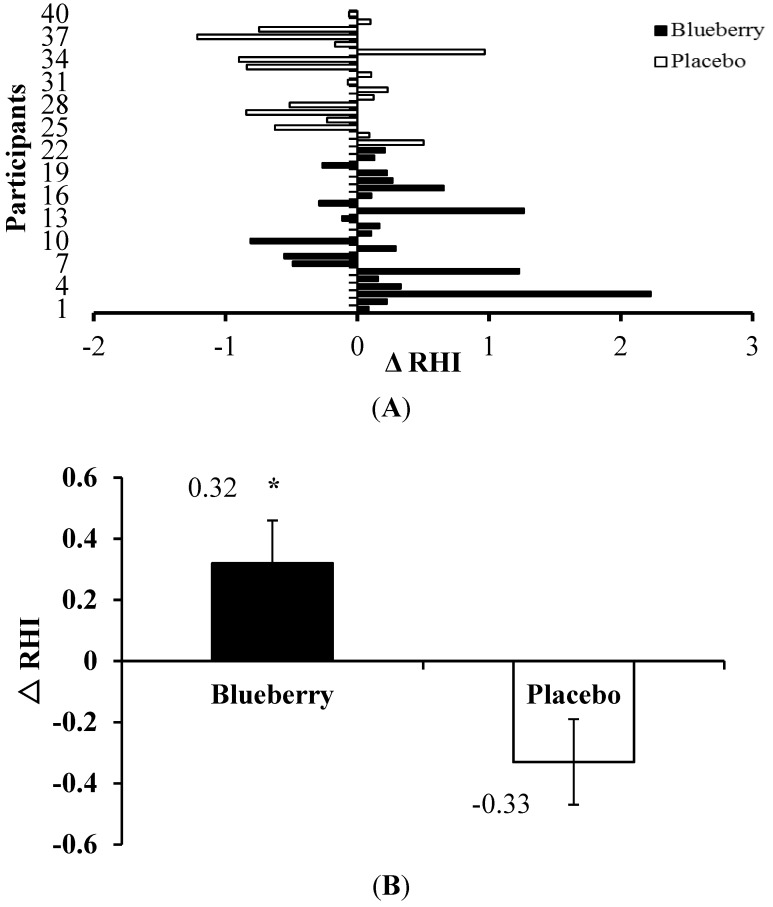
(**A**) Change in RHI (Δ; post- minus pre-intervention) in individual humans with metabolic syndrome who consumed the blueberry or placebo smoothie for six weeks; (**B**) mean change in RHI after adjusting for percentage of body fat and gender. * *p* = 0.0023 between groups (blueberry *versus* placebo). RHI, reactive hyperemia index (*i.e*., endothelial function). Values are the means ± SEM, *n* = 22 (blueberry) or *n* = 18 (placebo).

Forty participants (*n* = 22 blueberry; and *n* = 18 placebo) had complete pre- and post-intervention measurements for RHI (*i.e*., endothelial function). Although not significant, the average resting endothelial function increased in the blueberry group (pre-intervention, 1.94 ± 0.11 *versus* post-intervention, 2.17 ± 0.14), whereas it decreased in the placebo group (pre-intervention, 2.34 ± 0.16 *versus* post-intervention, 2.11 ± 0.15). However, comparison of the magnitude of changes between the groups revealed a greater effect of blueberry *versus* placebo in improving endothelial function (0.23 ± 0.14 *versus* −0.23 ± 0.13; *p* = 0.024). The majority (73%; 16 out of 22) of the blueberry group experienced a favorable (*i.e*., increased) change in endothelial function, whereas most of the placebo group (61%; 11 out of 18) demonstrated a decrease in this change during the six-week intervention period (Figure 2A). Even after adjusting for confounding factors, *i.e*., percent body fat and gender, the blueberry group still had a greater improvement in endothelial function when compared to their counterpart (Figure 2B).

**Table 2:** Baseline characteristics of participants with metabolic syndrome.

Variables	Blueberry	Placebo
	*n* = 23	*n* = 21
Race (African American/Caucasian), *n*/*n*	13/10	8/13
Gender (male/female), *n*/*n*	11/12	5/16
Age, years	55 ± 2	59 ± 2
Body weight, kg	100.8 ± 3.9	98.9 ± 3.0
BMI, kg/m^2^	35.2 ± 0.8	36.0 ± 1.1
Body fat, %	37.8 ± 1.4 *	42.8 ± 1.6
Fat mass, kg	38.5 ± 2.0	42.7 ± 2.3
Lean mass, kg	63.4 ± 2.9	56.8 ± 2.3
Clinic systolic blood pressure, mmHg	125.7 ± 2.2	125.0 ± 3.2
Clinic diastolic blood pressure, mmHg	82.7 ± 1.9	77.5 ± 1.9
Serum biochemistry		
Glucose, mmol/L	5.6 ± 0.1	5.6 ± 0.1
Insulin, pmol/L	129.3 ± 16.7	108.6 ± 9.1
Triglycerides, mmol/L	1.8 ± 0.2	1.4 ± 0.1
Cholesterol, mmol/L	5.4 ± 0.2	5.1 ± 0.2
LDL, mmol/L	3.2 ± 0.2	3.0 ± 0.2
HDL, mmol/L	1.3 ± 0.1	1.5 ± 0.1
24-h systolic blood pressure, mmHg ^#^	123.4 ± 1.9	124.7 ± 3.5
24-h diastolic blood pressure, mmHg ^#^	78.6 ± 1.2	76.1 ± 1.8
RHI ^#^	1.94 ± 0.11 *	2.34 ± 0.16
Antihypertensive medication users, %	91%	95%

Values are the means ± SEM; blood was drawn, and clinic blood pressure and RHI were measured from participants after a 10-hour fast; * blueberry *versus*. placebo, *p* ≤ 0.05. LDL, low-density lipoprotein; HDL, high-density lipoprotein; RHI, reactive hyperemia index (*i.e*., endothelial function); ^#^ RHI (blueberry, *n* = 22; and placebo, *n* = 18) and blood pressure (blueberry, *n* = 22; and placebo, *n* = 19).

**Table 3:** Changes in body weight, body composition, blood pressure and glucose, insulin and lipid concentrations of participants with metabolic syndrome.

Variables	Blueberry (*n* = 23)	Placebo (*n* = 21)
	Δ	Δ
Body weight, kg	0.9 ± 0.3	0.6 ± 0.3
BMI, kg/m²	0.3 ± 0.1	0.2 ± 0.1
Body fat, %	−0.1 ± 0.2	0.2 ± 0.2
Fat mass, kg	0.1 ± 0.2	0.4 ± 0.2
Lean mass, kg	0.5 ± 0.3	0.2 ± 0.3
Clinic systolic blood pressure, mmHg	−5.1 ± 3.0	−6.5 ± 2.4
Clinic diastolic blood pressure, mmHg	−5.5 ± 2.0	−7.3 ± 1.7
Serum biochemistry		
Glucose, mmol/L	0.1 ± 0.1	0.2 ± 0.1
Insulin, pmol/L	−6.6 ± 13.9	−2.9 ± 8.0
Triglycerides, mmol/L	−0.2 ± 0.1	−0.1 ± 0.1
Cholesterol, mmol/L	−0.9 ± 0.1	−0.6 ± 0.1
LDL, mmol/L	−0.6 ± 0.1	−0.3 ± 0.1
HDL, mmol/L	−0.2 ± 0.03	−0.2 ± 0.1
24-h systolic blood pressure, mmHg^#^	−1.1 ± 0.9	−0.9 ± 1.8
24-h diastolic blood pressure, mmHg^#^	−0.5 ± 0.5	−0.8 ± 0.7

Values are the means ± SEM. Blood was drawn, and clinic blood pressure was measured from participants after a 10-hour fast. Δ, change; post- minus pre-intervention. LDL, low-density lipoprotein; HDL, high-density lipoprotein. ^#^ Blood pressure, blueberry (*n* = 22) and placebo (*n* = 19).

## 4. Discussion

We found that increasing daily blueberry consumption in adults with metabolic syndrome for six weeks did not have a favorable effect on the blood pressure, a primary outcome for the clinical trial. However, endothelial function from baseline to end of trial was significantly improved, *i.e*., increased, despite not observing any changes in blood pressure. Thus, these findings were in agreement with Rodriguez *et al*. [20], who observed no changes in blood pressure after an acute dose of blueberries, but positive changes in endothelial function were noted in heathy men. Similarly, other clinical trials measured blood pressure with blueberry intake and also detected insignificant blood pressure findings in chronic smokers [22], healthy subjects with at least one risk factor for CVD [21] and obese insulin-resistant subjects [9]. On the contrary, clinical studies [19,23,25] have found significant decreases in systolic and/or diastolic blood pressures after consuming blueberries or anthocyanin-rich extracts for 8–12 weeks. These studies consisted of prehypertensive participants that also had metabolic syndrome (consumed 50 g/day freeze-dried blueberry powder) [25], were postmenopausal (also included stage 1 hypertension; consumed 22 g/day freeze-dried blueberry powder) [23] and had hypercholesterolemia (consumed 320 mg/day purified anthocyanin extracted from bilberry and blackcurrant) [19]. Compared to the previous studies [23,25], our study used a similar freeze-dried blueberry powder with a comparable dose of blueberries (45 g freeze-dried blueberry powder) and also a similar sample size of participants in each group.

There were study design differences that existed between the present and previous studies, which could have affected their differential outcomes on blood pressure. The previous studies had a single-blind study design with a non-matched placebo [25], a placebo that did not contain fiber [23,25] and an extract supplement *versus* whole food [19]. Not controlling for fiber could have potentially influenced the data given that fiber has been known to exert a protective effect on cardiovascular health [33]. The strength of our study was the double-blind, placebo-controlled and randomized study design. The present study’s blueberry smoothie had a matching placebo smoothie, and the two smoothies were similar in physical appearance, taste, macronutrient content (controlled for fiber) and consistency. We evaluated the synergistic effects resulting from consumption of whole freeze-dried blueberries (representing all of the bioactive compounds in the berry), rather than a berry extract consisting of specific bioactive compounds on CVD risk factors and endothelial function.

Polyphenol-rich foods, such as cocoa, green and black tea and soy, have been shown to provide significant benefits for endothelial function [34,35,36,37,38,39]. In our study, we evaluated the health benefits of blueberries, which are also rich in polyphenols, and an increase in endothelial function was observed. These findings also support the growing body of literature on blueberry consumption and endothelial function. Rodriguez *et al*. [20] found that an acute blueberry polyphenol intake improved flow-mediated dilation (*i.e*., assessment of endothelial function) in healthy men. Endothelial function had a time-dependent increase at 1–2 and 6 hours after consuming 766 mg of blueberry total polyphenols. After one hour of consuming various doses of blueberry total polyphenols, there was a dose-dependent increase in endothelial function up to the 766 mg intake followed by a plateaued endothelial function response with the higher doses. Furthermore, Zhu *et al*. found that acute and long-term (12 weeks) ingestion of a purified anthocyanin extract (320 mg anthocyanin/day) significantly improved flow-mediated dilation in hypercholesterolemic individuals [19]. In our study, the amount of anthocyanin and total polyphenol bioactives in the 45 g of blueberry powder were 581 mg and 1547 mg, respectively. In contrast to the current and previous findings, there were acute and six-week blueberry consumption clinical studies that demonstrated endothelial function was unaffected by consuming blueberries [21,24]. These discrepant findings on the effect of blueberries or anthocyanin extract intake on endothelial function may be due to the different doses of anthocyanin-rich berries consumed, processing of berries or assessment of endothelial function.

Elevated blood pressure has been shown to contribute significantly to endothelial dysfunction. High blood pressure causes functional alterations in the endothelium that are associated with decreased arterial mobility and increased stiffness in the arterial wall [40]. Our study found improvements in endothelial function with no significant changes observed in the blood pressure. Thus, it is possible that a longer study duration would have led to improvements in both endothelial function and blood pressure. Researchers have found that blueberries improved both blood pressure and endothelial function or a biomarker of endothelial function (*i.e*., nitric oxide) over a study duration of 8–12 weeks [19,23]. Furthermore, these improvements were not observed when evaluated mid-study. It is important to note that even though previous studies observed positive changes in both endothelial function and blood pressure with a longer duration than the current study, one consideration to explain our findings is that the majority of the participants in our trial were on antihypertensive medication, which was part of our inclusion criteria for metabolic syndrome. Our participants’ medication usage could have made their blood pressure levels closer to the lower end of the prehypertension range. The participants studied by Johnson *et al*. [23] had higher blood pressure values at baseline than our participants. However, the participants in the study by Zhu *et al*. [19] had similar blood pressure values as our participants, but they also used an anthocyanin extract *versus* the whole blueberry powder.

Additionally, our participants did not have any significant changes in body composition, fasting glucose, insulin and lipid levels, insulin sensitivity, and energy and macronutrient intake while consuming the blueberries for six weeks. These measurements, except insulin sensitivity, were also assessed in our previous clinical blueberry study, and similar null results were found [9]. It is possible that improvements in insulin sensitivity were not present in the current study because one may argue that a less precise method was used (*i.e*., FSIVGTT) *versus* the “gold standard” (*i.e*., hyperinsulinemic-euglycemic clamp). The clamp was used in our previous clinical blueberry study, which found that blueberries improved insulin sensitivity [9].

The smoothies in the current study contained milk and yogurt, and dairy products have been linked to a reduced risk of CVD in observational studies [41,42]. Although dairy products were added to both smoothies (*i.e*., blueberry and placebo) in our current and previous [9] studies, the group that consumed the blueberry smoothie displayed significant health benefits when compared to the group that consumed the placebo smoothie. Furthermore, it is possible that the food matrix of our blueberry smoothies influenced the antioxidant activity and bioavailability of the polyphenols. There is controversy about whether the proteins in milk interact with polyphenols and negate their antioxidant capacity and bioavailability. However, the data are still equivocal. The addition of milk to tea has been reported to inhibit the plasma antioxidant activity in humans [43]**,** whereas other researchers observed that adding milk to tea did not alter the plasma antioxidant activity in humans [44,45]. Reddy *et al*. [44] found lower levels of plasma catechins (*i.e*., polyphenol in tea) after participants consumed tea with milk compared to tea only. However, van het Hof *et al*. [46] observed that milk had no effect on plasma levels of catechins, and they concluded that milk does not affect the bioavailability of catechins. In regard to the food matrix effect of blueberries and milk, Cebeci *et al*. [47] found that the addition of milk decreased the total phenolic, flavonoid and anthocyanin contents of blueberries and also their antioxidant activity. However, the bioavailability of the total phenolic content was not affected with the addition of milk during an *in vitro* digestion simulation. The present study and our previous study used a blueberry smoothie that contained milk, which did not mask the beneficial effects of the blueberries on improving endothelial function and insulin sensitivity using the hyperinsulinemic euglycemic clamp [9]. Whether the addition of milk with polyphenol-rich foods impairs the bioavailability and antioxidant capacity of the polyphenols is still inconclusive, and further research needs to be conducted.

Our clinical trial also has several limitations. First, the serum biological markers of vascular function and plasma/urinary polyphenol metabolites were not analyzed in this study. Therefore, no mechanism(s) could be determined for the improvements in endothelial function. It has been inferred that the reduction in endothelial function is the result of a decrease in nitric oxide [48,49]. Thus, limited blueberry or anthocyanin extract studies have found that nitric oxide is involved with ameliorating endothelial function in humans [19,20,23]. Furthermore, an acute dose of blueberries has been found to improve endothelial function and was concomitantly linked to increased concentrations of plasma anthocyanins and phenolic metabolites [19,20]. Second, it is possible the participants’ blood pressure was near the lower range for prehypertension and that the consumption of blueberries would only provide a further minimal decrease in blood pressure. The majority of our participants were on antihypertensive medication, which could have contributed to their lower prehypertensive blood pressure levels. Furthermore, the participants’ medication dosage and usage were not standardized in the study, but we requested that the participants’ medication dosage and usage remained unchanged during the duration of the study. We used the World Health Organization criteria for metabolic syndrome, which included participants with elevated blood pressure and/or taking antihypertensive medication [2]. Finally, the unequal distribution of genders between the groups may have contributed to the differences in percent body fat between the groups. The placebo group consisted of more females and a higher percentage of body fat than the blueberry group. Although there were differences in percent body fat, consuming blueberries still increased endothelial function over a six-week period, and this remained significant, even after adjusting for percent body fat and gender.

## 5. Conclusions

In conclusion, six weeks of blueberry consumption on a daily basis did not improve blood pressure in a population with metabolic syndrome. Furthermore, the secondary outcome variable insulin sensitivity was unaffected. However, there was evidence of improved vascular health with the demonstration that endothelial function was improved (*i.e*., increased). This indicates that consumption of blueberries may increase endothelial function, even though blood pressure and other variables studied were not significantly affected. Although more validation trials are needed to fully evaluate this observation, our study does suggest a favorable benefit of blueberries on vascular health over a six-week period in adults with metabolic syndrome. Like most botanical and nutrition intervention studies, there is a substantial amount of inter-individual variability among the participants consuming the same dose of the botanical or nutritional product. Thus, future studies should evaluate the metabolomic and transcriptomic profiles of responders *versus* nonresponders to elucidate the different physiologic pathways that are affected by consuming blueberries. Furthermore, clinical trials with a longer duration (>8–12 weeks) evaluating the effects of blueberries or anthocyanin-rich foods on endothelial function and blood pressure prior to the study, mid-study and end of the study are warranted to explain the potential role in improving endothelial function and blood pressure in a population at high risk for developing CVD.
